# Sinus irrigation as an adjunctive therapy for odontogenic maxillary sinusitis — an in-depth analysis

**DOI:** 10.1186/s40902-024-00429-6

**Published:** 2024-06-11

**Authors:** M. Shriya Jaiswal, Gyu-Bong Ha, Ji-Young Hwang, Ja-Young Lee, Dae-Seok Hwang

**Affiliations:** 1 Department of Oral and Maxillofacial Surgery, Dental and Life Science Institute, Dental School, Pusan National University, Yangsan, 50612 Republic of Korea; 2 Dental Research Institute, Pusan National University Dental Hospital, Yangsan, 50612 Republic of Korea; 3 Department of Oral and Maxillofacial Surgery, Dental School, Pusan National University, Yangsan, 50612 Republic of Korea

**Keywords:** Odontogenic maxillary sinusitis, Sinus irrigation, Three-dimensional analysis, Iatrogenic sinusitis, Odontogenic infection, Peri-implantitis

## Abstract

**Background:**

Odontogenic maxillary sinusitis (OMS) is widely acknowledged in both the dentistry and otolaryngology fields. Recently, iatrogenic odontogenic maxillary sinusitis cases can be encountered frequently. The purpose of this study was to evaluate the effect of intraoral sinus irrigation using the small lateral window approach in patients with odontogenic maxillary sinusitis by comparing pre- and postoperative volumetric measurement of CBCT and symptoms.

We surveyed 21 patients who visited the Oral and Maxillofacial Surgery Department at PNUDH from 2016 to 2022. All the patients’ information was extracted from an electronic database. The patients with a follow-up period of 2 months or more were included. The three-dimensional volumetric measurement was performed using the ImageJ program (National Institute of Health, University of Wisconsin).

**Results:**

Among 21 patients, 16 (76.1%) were male, and 5 (23%) were female. The most common type of surgery was general anesthesia (16 cases) in which oroantral fistula was present in 7 cases. In the causes of maxillary sinusitis, there were seven implant-related patients, five patients of tooth extraction, seven patients of bone grafting, and two patients in other groups. Radiographic opacity decreased by 40.15% after sinus irrigation especially in bone graft and tooth extraction cases. Clinically, symptoms improved in 17 patients (80.9%).

**Conclusion:**

By this study, it can be concluded that maxillary sinus irrigation using the small lateral window approach is a clinically and radiologically effective treatment method for odontogenic maxillary sinusitis.

## Background

Odontogenic maxillary sinusitis (OMS) is a disease that accounts for 10–12% of all patients with maxillary sinusitis which can exceed to 41% [1–4]. In the case of conventional odontogenic maxillary sinusitis, it is mainly caused by periodontitis and odontogenic abscess, which are pathological conditions of the teeth and alveolar bone. The proportion of iatrogenic odontogenic maxillary sinusitis due to minor internal surgery is gradually increasing. In particular, due to the generalization of dental implant surgery and bone grafting using sinus elevation, the number of patients with unilateral maxillary sinusitis related to this is increasing [5, 6].

For patients affected by odontogenic maxillary sinusitis, antibiotic therapy serves as a common remedy. The microbial composition of OMS is diverse, encompassing both aerobic and anaerobic species during acute episodes. Addressing OMS involves antibiotic intervention as a crucial component within the treatment regimen, aiming to combat both aerobic and anaerobic bacteria. Despite antibiotic administration, OMS demonstrates resistance in approximately 79% of cases. The first-line antibiotic options for OMS include amoxicillin, amoxicillin-clavulanic acid, and clindamycin. Effective management relies on eradicating the infection source, which may entail a blend of medical and surgical interventions. Selection of the appropriate antibiotic is typically guided by prevalent microbiological patterns or findings from endoscopically directed cultures [3, 7, 8].

As a classical surgical method, Caldwell-Luc surgery can be performed by making a hole in the anterior part of the maxilla or the upper part of the ipsilateral second molar under general anesthesia and removing the pathological mucosa in the maxillary sinus. However, Caldwell-Luc surgery requires a long hospital stay, can cause bone defects in the outer and inner walls of the maxillary sinus, and can cause complications such as postoperative maxillary cyst (POMC) and inferior osteotomy blockade [4].

Maxillary sinus irrigation is a surgical procedure in which the side wall of the maxillary sinus is opened and washed with normal saline. In comparison to the conventional Caldwell-Luc procedure, sinus lavage has fewer complications, faster mucosal recovery, and is minimally invasive, so it can be performed under local anesthesia. In addition, the hospitalization period is short or temporary in some cases, so it has the advantage of less burden on patients and surgeons [9].

The diagnosis of maxillary sinusitis requires a detailed clinical and radiographic examination. The periapical and panoramic radiograph produces two-dimensional images and has certain limitations. Therefore, cone-beam CT scans are considered as a gold standard for sinusitis radiographic evaluation that gives high-resolution images in multiple planes and can also assess the anatomical structure, thickness, and volume of the maxillary sinus floor [6, 10, 11].

There have been many papers evaluating the effectiveness of Caldwell-Luc surgery in patients with unilateral maxillary sinusitis, but few papers have studied the effectiveness of sinus irrigation. Therefore, in this study, we aimed to evaluate the patients with odontogenic maxillary sinusitis who did not show improvement with antibiotic therapy, sinus irrigation using small lateral window was performed, and then the volume of the opaque image seen on cone beam CT was measured in three dimensions to evaluate the effectiveness of the procedure.

## Methods

### Study design and sample

The study group comprised a total of 21 patients who visited the Department of Oral and Maxillofacial Surgery, Pusan National University Dental Hospital, from January 2, 2016, to June 30, 2022. These patients with odontogenic maxillary sinusitis underwent sinus irrigation, and follow-up was done for at least 2 months. The choice of a follow-up period of 2 months can be influenced by several factors, and it is important to note that the specific duration may vary based on the severity of the sinusitis, the chosen treatment approach, and individual patient characteristics. The patients with maxillary sinusitis due to medication-related osteonecrosis of jaw (MRONJ), cysts, polyps, tumors, and infections were excluded. Informed consent from study participants was not required as none of their identity was revealed. This study was performed in accordance with the ethical standards of the Declaration of Helsinki and approved by Pusan National University Institutional Review Board (*IRB file no.: 2022-07-006*).

### Surgery

All 21 patients underwent sinus irrigation surgery, with 16 of them receiving treatment under general anesthesia. Nasotracheal intubation (NTT) was done. A vestibular incision or intrasulcular incision with vertical releasing cuts was performed at the respective locations, and periosteum was detached. During the procedure, a small lateral window was created. Conventionally, the thinnest lateral area below the infraorbital foramen is chosen in CBCT scans for this window placement as a standard practice. However, for this study, a slightly higher position of at least 10 mm above alveolar crestal bone was selected, and a window was created using 3-mm round bur, to proactively minimize potential future limitations in procedures like implant surgery or bone grafting. The inflammatory sinus membranes and foreign bodies were removed, and the sinus cavity was irrigated before being packed to establish hemostasis. Later, antibiotics were prescribed to all of the patients. The maxillary sinus irrigation was done with either saline or iodine solution. The postoperative symptoms were assessed.

### Methods

#### Measurement of the volume of the opaque area in the maxillary sinus

**Fig. 1:**
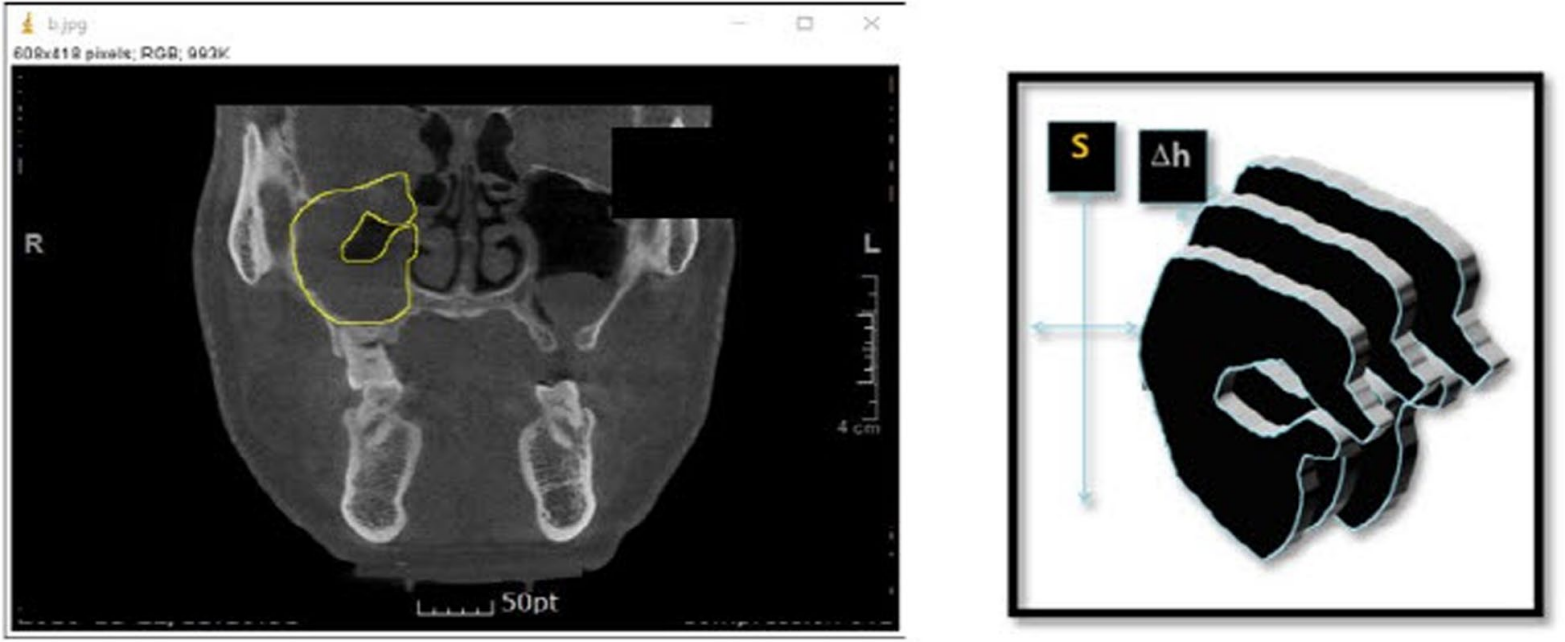
This figure shows the measurement of surface using ImageJ program (right) and the method to measure the volume of maxillary sinus using CT images (left)

Images taken with cone beam CT (*ProMax, Planmeca, Finland*) were analyzed. The scanner parameters were as follows: 20 × 19 cm field of view, 110 kVp, 4.0-mA tube current, and 24-s scan time. The three-dimensional volume measurement of the radiopaque area as shown in image was measured using the ImageJ program provided by the National Institute of Health (NIH). The area (S) was evaluated by measuring the number of opaque pixels visible in each image using the ImageJ program (Fig. 1). The slice thickness was kept to be 1 mm.

The volume (V) of the opaque area was obtained by multiplying the measured area (S) by the thickness (h) of the slice. The volume of the opaque area in the entire maxillary sinus was calculated by adding the volumes of all slides.

The degree of progression of maxillary sinusitis was evaluated by calculating the volume ratio of the opaque area to the total sinus volume (Vs). The volume of the opaque image in the maxillary sinus before surgery was designated as Vt0, and the volume of the opaque image after sinus irrigation was designated as Vt1.

$$V =\Sigma (\mathrm{S }\times \mathrm{ h})$$

*V*: volume, S: surface, h: slice depth

$$\mathrm{Maxillary\ sinusitis\ value }= {{\text{V}}}_{{\text{t}}0}/{{\text{V}}}_{{\text{s}}}$$

### Statistical analysis

The statistical software SPSS (*ver. 16.0, SPSS Inc., Chicago, IL, USA*) was used for statistical analysis. The age of males and females were compared using independent sample *t*-test. The chi-square test was done to find the association between patients and causes of maxillary sinusitis. A *p*-value of < 0.05 was considered statistically significant.

## Results

### Patients and demographic data

A total of 21 patients were included for this study. Out of which, 16 (76.1%) were male, and 5 (23.8%) were female with the mean age of 53.71 ± 15.27 years (range 26–80 years). The mean ages of males and females were 51.9 ± 14.1 (range 26–76) years and 59.4 ± 19.1 (range 34–80) years respectively. The difference between the mean ages of males and females was not statistically significant (*p* = 0.8). A total of 7 (33.3%) patients had an oroantral fistula, and maxillary sinus irrigation was performed in 16 (76.1%) patients under general anesthesia and 3 (14.2%) outpatients under local anesthesia (Table 1).

**Table 1:** Demographic data of patients with maxillary sinusitis

	**Male**	**Female**	**Total**
**Age**			
Mean	51.9 ± 14.1	59.4 ± 19.1	53.71 ± 15.27
Range	26–76	34–80	26–80
**Anesthesia**			
General	12	4	16
Monitored	2	-	2
Local	2	1	3
**Oroantral fistula**			
Positive	3	4	7
Negative	11	3	14
**Total**	16	5	21

### Etiology of maxillary sinusitis

Regarding the etiology of maxillary sinusitis, of total 21 patients, 7 (33.33%) patients with implants were included; three of the above cases resulted from maxillary sinus perforation during implant placement, and three others from sinus fistula following implant removal. One case of peri-implantitis was reported. Five (23.8%) patients experienced it following tooth extraction, as a result of an oral maxillary sinus fistula. After maxillary sinus augmentation and bone grafting, sinus infections were the most frequent cause (7 patients — 33.33%). Other causes (2 patients — 9.52%) included the invasion of the maxillary sinus, fracture of the sinus’s side wall, invasion of the fixed metal plate into the sinus following open reduction and internal fixation, and invasion of the maxillary sinus caused by materials used in endodontic treatment. According to the chi-square test, there was no correlation (*p* = 0.74) between the etiology of maxillary sinusitis and the patients (Table 2).

**Table 2:** Etiology of maxillary sinusitis

Causes	Patient
1. Implantation	
a. Implant placement	4 (19.0%)
b. Fixture removal	2 (9.50%)
c. Peri-implantitis	1 (4.76%)
2. Tooth extraction	5 (23.8%)
3. Bone graft	7 (33.33%)
4. Others	2 (9.52%)
**Total**	**21**

### Effect of sinus irrigation on the opaque area of the maxillary sinus

**Fig. 2:**
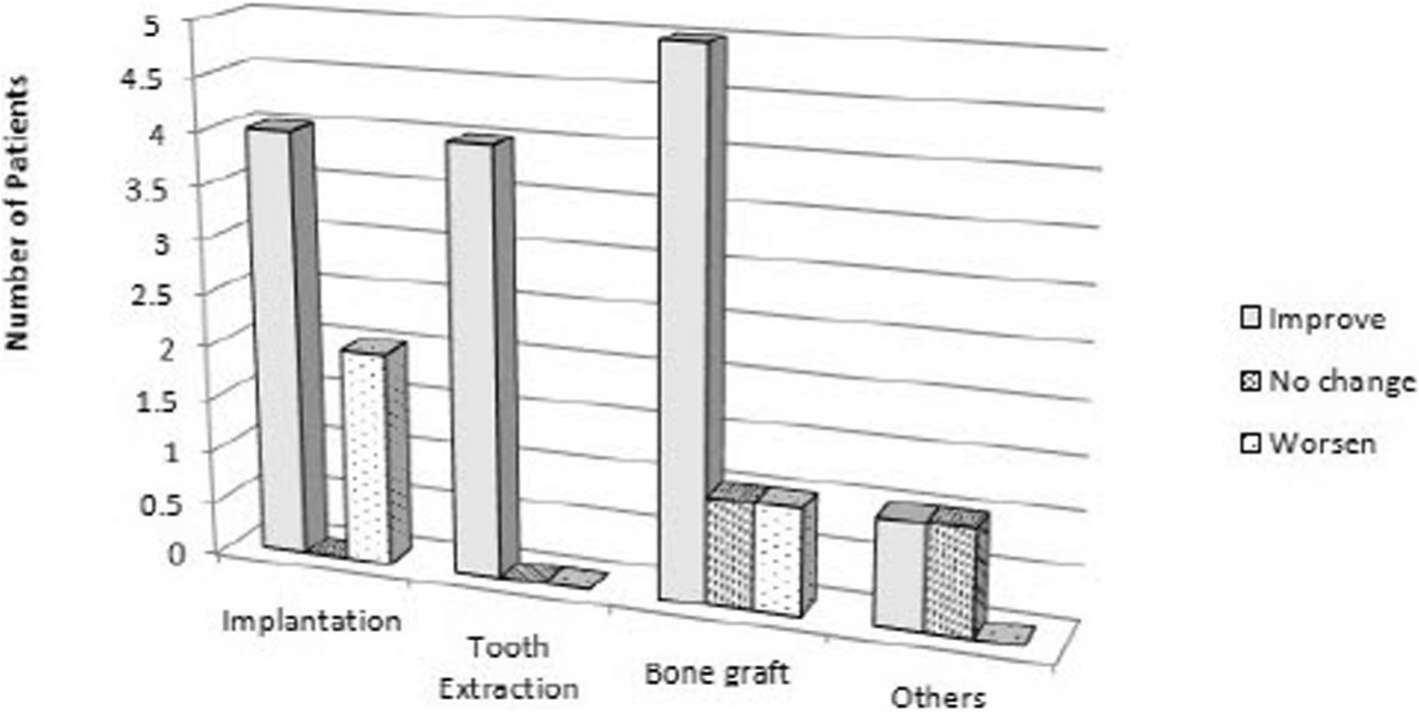
Radiographic haziness by causes

Five (71.4%) of the seven patients in Fig. 2 who had odontogenic maxillary sinusitis as a result of implantation showed improvement because of decreased opacity, while two (28.5%) patients showed increasing radiopacity. Following tooth extraction, all five (100%) patients who had maxillary sinusitis had less opacity. Additionally, after sinus irrigation, 5 (71.4%) out of 7 patients who received maxillary sinus elevation and bone graft experienced a decrease in radiopacity, whereas 1 patient experienced a rise in opacity. The opaque area’s volume did not change for the other group.

Patients with metal fixation plates showed no change, while those with maxillary sinusitis brought on by endodontic treatment materials had decreased opaque areas. Out of the 21 patients, 16 (76.1%) experienced a decrease in the radiopaque image volume following sinus irrigation, 2 experienced no change, and 3 experienced an increase. The results of the chi-square test indicated that there was no significant difference (*p* = 0.29) between the etiology of maxillary sinusitis and the degree of improvement.

### Effect of sinus irrigation on clinical symptoms

**Fig. 3:**
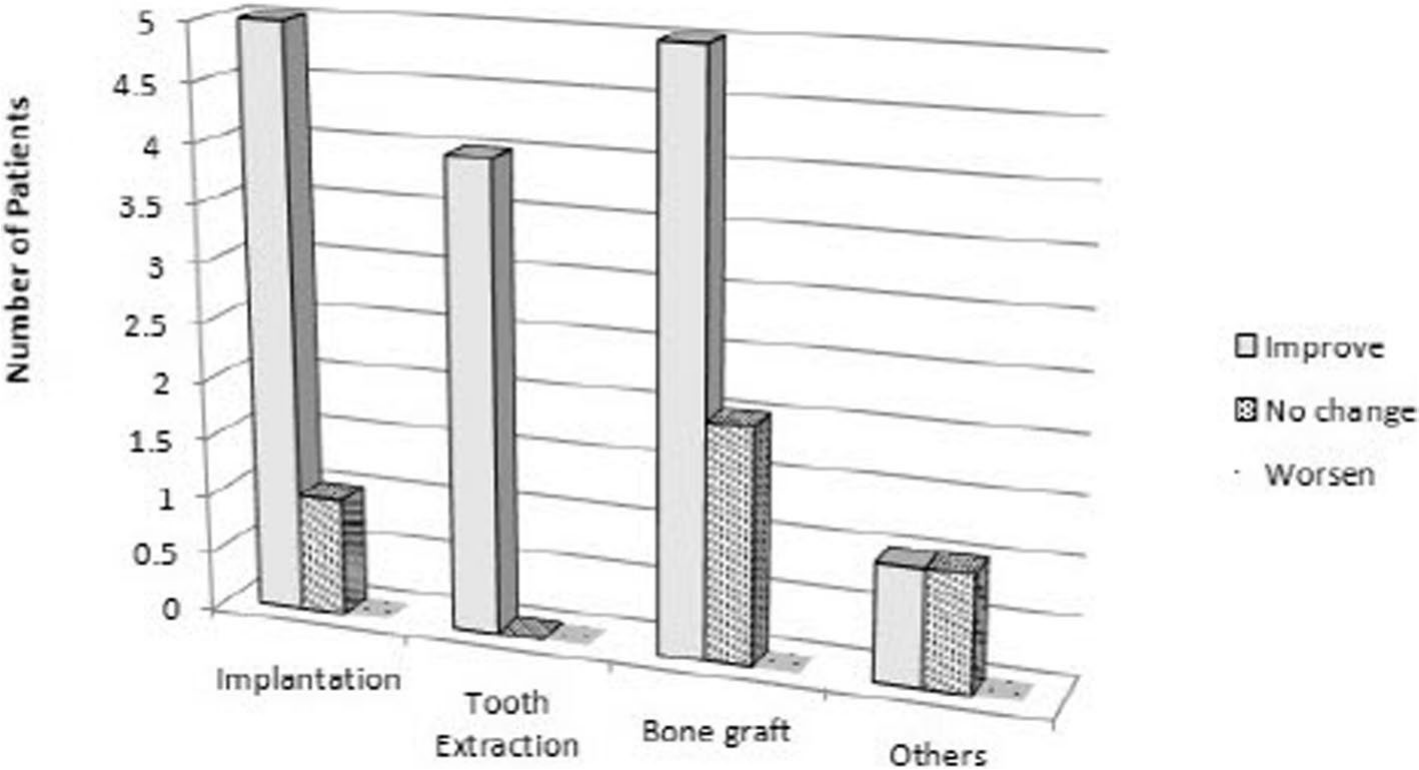
Clinical symptom by causes

Of a total of 21 patients, six (85.7%) of the seven implant patients in Fig. 3 reported improved clinical symptoms following surgery, whereas one (14.2%) patient had postoperative face pain. All five patients (100%) who had tooth extractions saw an improvement in their clinical symptoms. After having a sinus lift surgery, 5 (71.4%) of the patients in the group with maxillary sinusitis reported a reduction in symptoms, while 2 (28.5%) continued to have discomfort. While patients with metal fixation plates did not exhibit any reduction in symptoms, those receiving endodontic therapy in other patient groups did show improvement in their clinical symptoms. Seventeen (80.9%) out of 21 patients, excluding 4 (19%) patients, showed improvement in clinical symptoms, and there was no patient whose symptoms deteriorated.

### Changes in opacity volume pre and post maxillary sinus irrigation

**Fig. 4:**
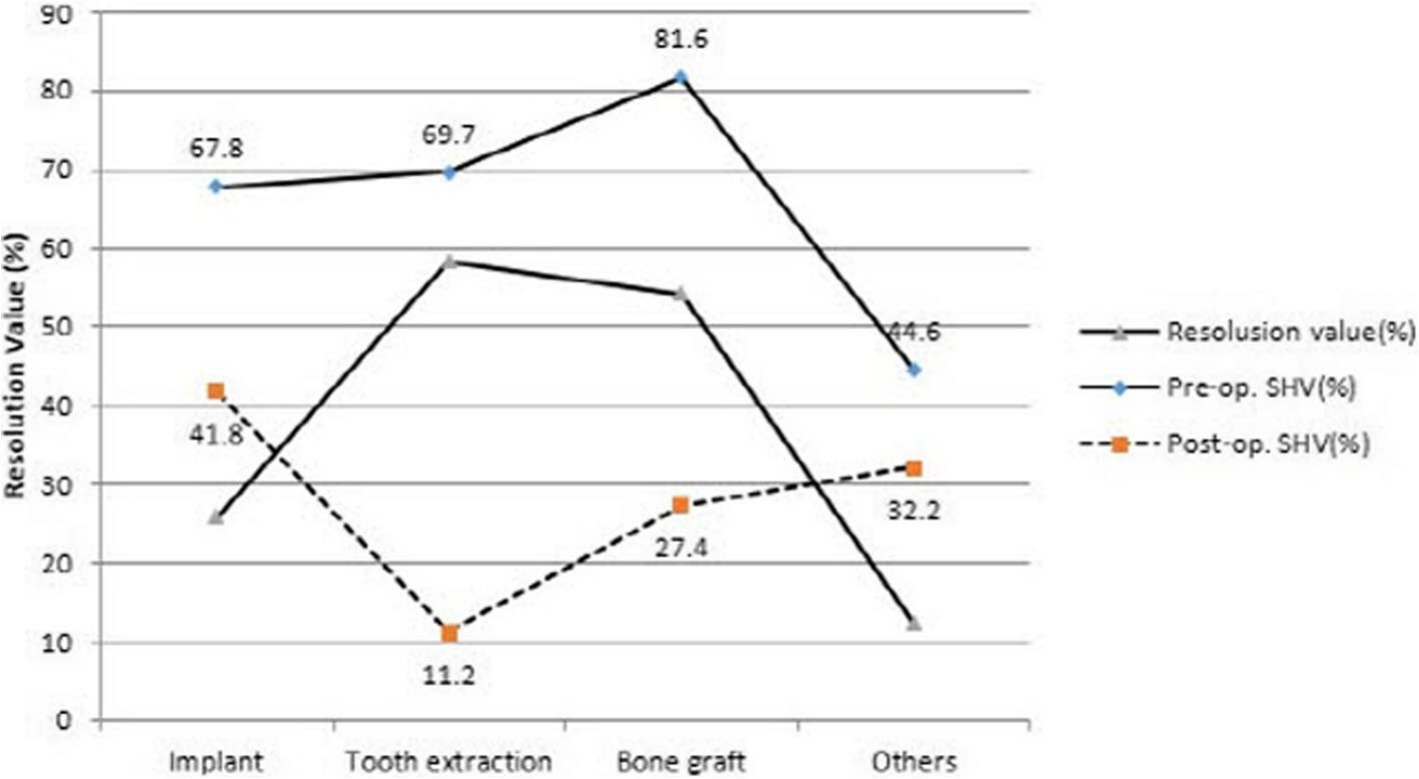
Mean resolution rate of maxillary sinusitis

The volume ratio of the radiographic image before maxillary sinus irrigation is depicted in Fig. 4 by the solid line. Regarding patients with implant-related maxillary sinusitis, the radiopaque image accounted for roughly 67.8% of the sinus volume. Approximately, 69.7% of the total sinus volume displayed radiopaque images in the tooth extraction cases. The patients with the highest volume ratio, representing 81.6% of the opaque image, were those who had bone grafting following maxillary sinus elevation. At 44.6%, the other patient group had the lowest volume ratio. The volume of the opaque image to the total sinus volume following sinus irrigation is indicated by the dotted line. Patients who had their teeth extracted had the greatest drop in the opaque image’s volume ratio following surgery—11.2%. After surgery, the patient group whose maxillary sinus lift was the cause displayed a volume ratio of 27.4%, whereas the percentage dropped to 41.8% in patients whose cause was the implants and showed the smallest reduction ratio of 32.2% in patients with other causes.

## Discussion

Maxillary sinusitis (acute or chronic) is defined as a symptomatic inflammation of the maxillary sinus, usually caused by viral, bacterial, allergic, or fungal rhinitis [12]. However, any disease arising from dentoalveolar structures could damage the floor of the maxillary sinus leading to a sinusitis known as odontogenic maxillary sinusitis (OMS). The OMS is a well-recognized but understudied form of sinusitis that requires a unique treatment regimen that differs from non-odontogenic sinusitis [1].

The diagnosis of odontogenic maxillary sinusitis should be done systematically using dental examinations, radiographs, and other 3D radiographic modalities [6]. Rosenfeld et al. in his literature described CBCT as a gold standard for radiographic evaluation of the paranasal sinuses [13]. Bomeli and Matsumoto et al. concluded that unilateral maxillary sinusitis is the common radiologic finding of odontogenic sinusitis [14, 15].

The mainstay of treatment for odontogenic sinusitis is surgical therapy, and odontogenic sinusitis is often resistant to trials of antibiotics. Antibiotics, however, do play a role whenever combined with other appropriate treatments. Odontogenic sinusitis patients have a larger and more diverse microbiological burden than that observed in chronic rhinosinusitis alone, and antimicrobial therapy should address this difference [16]. Broad coverage of polymicrobial and anaerobic populations can often be achieved with a penicillin (amoxicillin) and a beta-lactamase inhibitor, with or without metronidazole [10]. Saibene et al. showed that 70% of odontogenic sinusitis isolates were susceptible to amoxicillin clavulanate, and 80% of the *Staphylococcus* spp. cultured were capable of producing beta-lactamase [17]. And for individuals with a penicillin allergy who cannot receive amoxicillin, doxycycline is the most appropriate treatment.

Within this study, all patients received antibiotic medication for 1 week. If symptoms improve after about 1 week of medication, administer medication for another week and follow up. If there is no improvement in symptoms, surgical treatment such as removing the odontogenic cause and irrigation of the maxillary sinus can be selected. After the dental cause is removed, primary closure is performed, and maxillary sinus irrigation is done as sinus irrigation can relieve postoperative reactions, reduce the probability of sinus infection, and protect the function of the maxillary sinus [9, 18].

To investigate the effect of maxillary sinus lavage, the volumes of opaque images in the sinus observed on CBCT were compared before and after sinus lavage. According to John’s report, who conducted a literature review, the average age of patients infected with odontogenic maxillary sinusitis was about 51.2 ± 3.9, and the age ranged from 43 to 58 years. The mean ages of males and females were 49.4 ± 10.8 years (range 33–67) and 50.6 ± 10.8 (range 33–67) years respectively [19]. According to our results in Table 1, out of 21 patients, 16 were male, and 5 were female. So, it can be said that there were more male patients than females. The age distribution ranged from 26 to 80 years, and the average age was 53.71 ± 15.27 years. The difference between the mean ages of males and females was not statistically significant. At a young age, many cases of oral disease can lead to an odontogenic disease, and minor surgeries such as tooth extraction or implantation are rare, so it is thought to be most prevalent in the middle age.

According to a paper published by Matthias in 2015 based on 174 cases, oroantral fistula after tooth extraction was the most common cause with 60 cases (34.5%), followed by peri-implantitis in 9 cases (5.2%). Sinus lift was the cause in four cases (2.3%), which accounted for a relatively low proportion [20]. Contrary to this, according to Kim et al., out of 27 patients, 10 patients (37%) had maxillary sinusitis due to implant, and 8 patients (29.6%) had maxillary sinusitis due to tooth extraction [8]. In Table 2, it is shown that the causes of dental maxillary sinusitis were related to implant placement; removal and peri-implantitis in 7 cases (33.3%); bone graft in 7 cases (33.3%), which accounted for the most proportion; and tooth extraction in 5 cases (23.8%).

Several causes may contribute to implant failure. In a prior investigation into the late stages of chronic sinusitis-related implant failure, it was found that long implant apex perforation into the sinus, microbe infection, and contamination of potentially toxic elements may be major factors in dental implant failure linked to maxillary sinusitis [21, 22]. According to Kim et al. case report, who performed lateral sinus irrigation on two patients with maxillary sinusitis after implant surgery, it was observed that all opaque images on cone beam CT decreased significantly 2 weeks after sinus irrigation [23]. According to Fig. 2, opaque images on radiographs improved in most cases after maxillary sinus irrigation. However, in some patients with maxillary sinusitis caused by implant placement (may due to severity of peri-implantitis or degree of osseointegration) and bone grafting, opacity increased because the bone particles were not easily washed away and remained at its position [24].

Gang et al. showed that the chronic maxillary sinusitis was healed within 8–12 weeks of Caldwell–Luc surgery followed by sinus irrigation. None of the patients complained about nasal obstruction or facial paresthesia, and no further major complications were noticed after 36 months of surgery [9]. In our study, Fig. 3 shows the change in clinical symptoms after maxillary sinus irrigation. It was found that the symptoms improved in 17 out of 21 patients, and no patient’s condition jeopardized.

Bomeli et al. in his literature found that around 79% of sinuses had opacity, and its cause was unknown dental source [14]. Likewise, Maillet et al. found that 72.5% had odontogenic cause, whereas 27.5% had unknown dental cause [25]. However, according to our study, as shown in Fig. 4, the radiopacity of maxillary sinusitis patients was highest in bone transplant patients (81.6%). The effect of maxillary sinus irrigation was the greatest in extraction patients and the least in implant patients except for others.

Therefore, we can say that sinus irrigation through lateral window approach is an effective treatment method. However, the few limitations of this study were retrospective nature, small number of patients, and long-term follow-ups. Therefore, further study should be done to generalize our results.

## Conclusion

In this study, patients who underwent sinus irrigation showed a decrease in sinus permeability, and the reduction amount was about 40.15% on average. Clinical symptoms were improved in 17 (80.9%) out of 21 patients, and symptoms were still present in 4 patients (19%).

Therefore, sinus irrigation through lateral window approach is an effective treatment method clinically for patients with acute odontogenic sinusitis caused by iatrogenic disease.

## Data Availability

All data generated or analyzed during this study are included in this published article.

## Abbreviations

OMS: Odontogenic maxillary sinusitis
CBCT: Cone beam computed tomography
POMC: Postoperative maxillary cyst
